# *Staphylococcus aureus* Toxins: Promoter or Handicap during Infection?

**DOI:** 10.3390/toxins13040287

**Published:** 2021-04-19

**Authors:** Bettina Löffler, Lorena Tuchscherr

**Affiliations:** Institute of Medical Microbiology, Jena University Hospital, 07747 Jena, Germany; Lorena.TuchscherrdeHauschopp@med.uni-jena.de

*Staphylococcus aureus* is an opportunistic and versatile pathogen that can cause several diseases, which range from acute and destructive, to chronic and difficult-to-treat infections [1,2]. *S. aureus* colonizes the nasopharynx of many individuals, but this colonization can be the source for infections [3] that vary from superficial mild skin infections to severe diseases, such as pneumonia or osteomyelitis [4,5]. In particular, severe staphylococcal infections are associated with significant morbidity and mortality. The ability of *S. aureus* to trigger different types of infection is due to its wide repertoire of virulence factors and infection strategies [2,6,7].

To initiate the infection, *S. aureus* uses different surface-bound proteins that facilitate the pathogen to attach to host tissue and invade host cells [8,9,10]. Following adherence and internalization, the bacteria can grow and activate their toxin production largely controlled by global regulators, such as quorum sensing systems [11]. *S. aureus* can express many molecules, in particular toxins, that harm and destroy host cells [12,13,14,15,16]. During the infection process, the toxins enable the bacteria to destroy and enter deep tissue structures, to obtain nutrition for their growth, and to defend against the immune system [16]. However, after the acute and destructive phase, *S. aureus* bacteria aim for survival within the host [17,18]. For bacterial long-term persistence in host tissue, many of the toxins need to be downregulated. In this way, the bacteria are hardly recognized by the immune system and can avoid clearance by phagocytes. Consequently, downregulation of toxins is crucial to silently persist within host cells/tissue for long time periods [18,19,20].

Taken together, virulence factors, such as toxins, need to be regulated precisely during the course of infection by global regulators, e.g., quorum-sensing systems, which act as a feedback to the surrounding microenvironment [21,22,23,24,25]. Even though staphylococcal toxins are deeply studied, the question remains as to whether and at which stage of the infection a “virulent” strain which expresses a lot of secreted toxins, or a “silent” persisting strain is the real danger for the host.

The purpose of this Special Issue is to publish original research and review articles related to the role of toxins in disease development, the mechanisms of toxin effects in host tissue, the regulation and function of toxins during acute and chronic infections, and toxins as targets for vaccine development and therapeutic interventions. A better understanding of the role of toxins during the different stages of infection may enable us to precisely plan anti-toxic drugs/vaccinations or therapeutic interventions in bacterial regulation.

In two reviews, the importance of *S. aureus* enterotoxins and exotoxins as foodborne intoxications are addressed. The first review highlights the role of staphylococcal enterotoxin C in human and animal health, whereas the second review focusses on measures to avoid food poisoning originating from mastitis or skin infection from ruminants [26,27]. For this, rapid methods to detect *S. aureus* exotoxins in dairy products are required.

The function and effects of toxins that are major virulence factors in serious invasive infections are outlined in the next section. Two original research articles deal with *S. aureus* leukocidins that efficiently induce cytotoxicity in susceptible leucocytes, whereas these effects are highly cell type- and species-specific. In the article by Hodille et al., the effect of leukocidins on bone marrow cells is analyzed to better understand leukopenia, which is described in many clinical cases [28]. In the work by Tromp et al., the host cellular pathways that are activated after the binding of the leukocidins to the cellular receptor of susceptible cells are elucidated [29]. The authors describe post-translational modification pathways that could explain differences in the susceptibility to leukocidins. In a further contribution, the production and release of *S. aureus* extracellular vesicles (EVs) that are packed with different cytotoxic components and represent a novel bacteria secretory system in response to various stress conditions are analyzed [30]. Finally, the article by Schelin focusses on bacterial regulator networks that control the expression of *S. aureus* toxins, e.g., superantigens. The authors performed RNAseq analysis, and revealed the role of ClpXP in the complex regulation of *S. aureus* virulence [31].

Two research papers within this Special Issue analyzed the role of toxins in the development of *S. aureus* pneumonia. Deinhardt-Emmer et al. demonstrated that pneumonia strains exhibit enhanced invasion and cytotoxicity, whereas when the strains were obtained from a viral–bacterial co-infection, these virulence characteristics were not required [32]. Lacoma et al. characterized *S. aureus* isolates from mechanically ventilated patients. Although the authors performed an extensive analysis, there was no evidence of pathological adaptation related to virulence, resistance, or niche adaptation [4]. From these two papers, we can conclude that almost any *S. aureus* strain is able to cause a lung infection when the lung is already damaged, e.g., by a preexisting influenza infection or by a mechanical ventilation.

Chronic and difficult-to-treat *S. aureus* infections are major clinical problems which are addressed in this Special Issue as well. One example is chronic rhinosinusitis, and the *S. aureus* leucocidin ED ((LukED) is described as a constant trigger that contributes to disease development [33]. A second well-known disease that is complicated by *S. aureus* infections is cystic fibrosis (CF). A prospective multicenter study followed the adaptation of *S. aureus* isolates in CF patients. The authors described a decrease in virulence and toxin genes that most likely reflect the bacterial adaptation process towards a persisting phenotype [34]. Additionally, in *S. aureus* osteomyelitis, the bacteria revealed adaptation strategies largely controlled by the accessory gene regulator (*agr*) locus, which is extensively reviewed by Butrico et al. [35]. Finally, Wong Fok Lung et al. display the *S. aureus* metabolic adaptation that is induced by the host immune response and results in bacterial strains adapted for chronicity [36].

We conclude the Special Issue on *S. aureus* toxins with two manuscripts addressing prophylactic and therapeutic interventions. Shukla et al. propose a statistical method to integrate data on changes in gene expression upon antimicrobial treatments. This method aims to develop a therapeutic regime to not only eliminate bacteria, but also reduce their virulence [37]. Joyner et al. developed a virus-based vaccine that target *S. aureus* toxin (Hla) and attenuates *S. aureus* Hla-mediated pathogenesis [38]. This approach could be part of a multi-component *S. aureus* vaccine in future.
